# Task Offloading and Resource Allocation Strategy Based on Deep Learning for Mobile Edge Computing

**DOI:** 10.1155/2022/1427219

**Published:** 2022-08-31

**Authors:** Zijia Yu, Xu Xu, Wei Zhou

**Affiliations:** School of Information Engineering, Suzhou University, Suzhou, Anhui 234000, China

## Abstract

For the problems of unreasonable computation offloading and uneven resource allocation in Mobile Edge Computing (MEC), this paper proposes a task offloading and resource allocation strategy based on deep learning for MEC. Firstly, in the multiuser multiserver MEC environment, a new objective function is designed by combining calculation model and communication model in the system, which can shorten the completion time of all computing tasks and minimize the energy consumption of all terminal devices under delay constraints. Then, based on the multiagent reinforcement learning system, system benefits and resource consumption are designed as rewards and losses in deep reinforcement learning. Dueling-DQN algorithm is used to solve the system problem model for obtaining resource allocation method with the highest reward. Finally, the experimental results show that when the learning rate is 0.001 and discount factor is 0.90, the performance of proposed strategy is the best. Furthermore, the proportions of reducing energy consumption and shortening completion time are 52.18% and 34.72%, respectively, which are better than other comparison strategies in terms of calculation amount and energy saving.

## 1. Introduction

With the rise of computing-intensive applications and explosive growth of data traffic, users' requirements for the computing power and service quality of mobile devices are also increasing [1]. At present, cloud computing also faces many problems and challenges. Due to its resource-intensive architecture, mobile cloud computing imposes a huge additional load on the backhaul link of mobile networks [2, 3]. Thus, Mobile Edge Computing (MEC) technology is proposed, which physically integrates computing and storage resources into the edge of mobile network architecture [4, 5]. This not only effectively reduces the transmission delay but also solves the problems of high load and high delay caused by mobile cloud computing [6]. At the same time, MEC has the characteristics of distributed architecture, being at the edge of network, low latency, user location awareness, and network status awareness [7, 8]. However, deploying a large number of computing and storage devices at the edge of network for users to choose and accessing neighboring service providers for edge computing will bring a series of complexities such as access and resource allocation strategy selection, user mobility management, and computing task migration problems [9].

In order to achieve the goal of short completion time and lower terminal energy consumption under delay constraint, this paper proposes a task offloading and resource allocation strategy based on deep learning for MEC. In order to shorten the completion time of computing tasks and minimize the energy consumption of all terminal devices while satisfying delay constraints, the proposed strategy is designed in a multiuser multiserver MEC environment, combined with computing model and communication model in system. Moreover, a new objective function is designed, which uses objective optimization to further reduce energy consumption and time delay. It uses Dueling-DQN algorithm to solve the optimization model to shorten completion time and minimize energy consumption of all terminal devices while meeting the delay constraints.

The remaining chapters of this paper are arranged as follows: Section 2 introduces the relevant research work on mobile task unloading. Section 3 introduces the system model. Section 4 introduces the new computing offload method based on improved DQN. In Section 5, simulation experiments are designed to verify the performance of the proposed model. Section 6 is the conclusion.

## 2. Related Work

In MEC network, computation offloading may occur in three types: full offloading, partial offloading, and local processing [10]. An important research hotspot in the field of computation offloading is computation offloading decisions. Generally speaking, the offloading goal mainly focuses on minimizing the overall delay or minimizing energy consumption for user devices while meeting the minimum delay requirements [11]. Reference [12] proposed a distributed task offloading strategy for low-load base station groups in MEC environment. It selects the best MEC node offloading amount by game equation on the basis of quantifying offloading cost and delay. But it is not suitable for high-load application environments. In reference to the problem of unbalanced computing resources on the edge server in vehicle edge computing network, [13] proposed a load balancing task offloading scheme based on software-defined network. This solution can effectively reduce delay and improve the efficiency of task offloading processing. However, the processing method used has poor performance, which affects the distribution efficiency. Reference [14] used greedy selection to design a maximum energy-saving priority algorithm to achieve optimal offloading of computing tasks on mobile devices, but it does not consider the delay constraints of task offloading. Reference [15] combined long short-term memory (LSTM) and candidate network set to improve the deep reinforcement learning algorithm and used this algorithm to solve the problem of offloading dependency of multinode and mobile tasks in large-scale heterogeneous MEC. But they ignore the optimal allocation problem of computing resources.

Similar to computation offloading, resource allocation is also one of the core issues in MEC [16]. In MEC network, technologies such as content caching and ultradense deployment are introduced, and multiple resources are deployed to mobile network edge according to the specific needs of users. This can further ensure the quality of service and greatly increase system capacity [17]. However, due to objective reasons such as physical volume and power consumption, the mobile network edge has limited computing resources, storage cache capacity, and spectrum resources. How to allocate multiple resources and improve the system service efficiency has a huge effect on the improvement of MEC network system performance [18]. Reference [19] proposed a time average computation rate maximization (TACRM) algorithm, which allows joint allocation of radio resources and calculation resources. However, the overall performance and task requirements of devices were not considered comprehensively in the allocation process, and the allocation efficiency still needs to be improved. Reference [20] comprehensively considered factors such as CPU, hard disk space, and required time and distance and proposed a comprehensive utility function for MEC resource allocation to achieve the optimal allocation of resources in MEC and cloud computing. However, this function considers many factors, which will seriously affect the efficiency of allocation in real applications. Reference [21] designed a two-layer optimization method for MEC, which uses pruning candidate modes to reduce the number of unfeasible offloading decisions. Through ant colony algorithm to achieve the upper-level optimization, the resource allocation effect is better. However, the server computing resource constraints and task delay constraints are not considered, and the overall timeliness is not good. Reference [22] constructed a low-complexity advanced branch model, which can be used for resource scheduling in large-scale MEC scenarios.

Due to the lack of powerful processing algorithms, the overall efficiency and performance are not ideal. To this end, comprehensively considering the task offloading and resource allocation problem, a deep learning-based MEC task offloading and resource allocation strategy is proposed to coordinate and optimize the allocation and offloading between computing resources and computing tasks, which improves the comprehensive computing efficiency of MEC.

## 3. System Model

**Figure 1:**
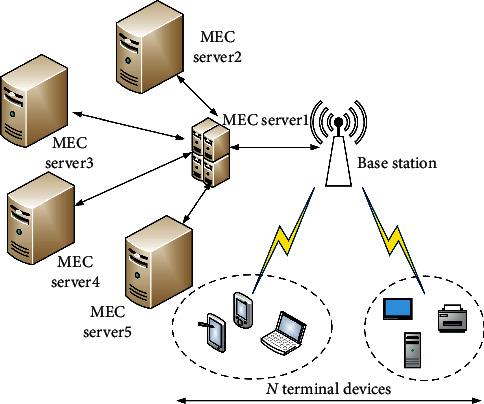
System model diagram.

The system model is a multiuser multiserver application scenario, in which there are *N* terminal devices and *M* MEC servers. The base station is used to provide communication resources for user equipment. Each base station is connected to an edge computing server through optical fiber, through the wireless communication link to connect to MEC server to calculate task data of offloading terminal devices, as shown in Figure 1. It is assumed that each terminal device can perform offloading computations or local calculations for its own execution tasks. And when offloading, the task can only be offloaded to one MEC server for calculation, and each terminal device is within the range of wireless connection. However, the computing power of each MEC server is limited; it cannot accept the offloading request of each terminal at the same time.

The collection of terminal devices is *U*={1,2,…, *n*,…, *N*}, the collection of MEC server is *H*={1,2,…, *m*,…, *M*}, and the collection of all tasks is *G*. Each terminal device *n* has a calculation-intensive task *G*_*n*_ to be processed, which specifically includes the data *D*_*n*_ (code and parameters) required for computing task *G*_*n*_, the CPU workload *ϕ*_*n*_ required for computing task *G*_*n*_, and the completion time of task *G*_*n*_. The extension constraint is *τ*_*n*_, namely, *G*_*n*_≅(*D*_*n*_, *ϕ*_*n*_, *τ*_*n*_). The set of offloading decisions for each *G*_*n*_ is *X*=[*x*_1_, *x*_2_,…, *x*_*n*_,…, *x*_*N*_].

When *x*_*n*_={0,1,…, *m*,…, *M*}, and *x*_*n*_=0 is local offloading, the rest means offloading *G*_*n*_ to *m* MEC servers.

### 3.1. Communication Model

In the computation offloading problem, two links are mainly studied: wireless link from terminal devices to MEC and the wired link from MEC to cloud in the core network. In the wireless link, Finite-State Markov Channel (FSMC) model based on fading characteristics is used. FSMC model has a wide range of applications in wireless networks [23, 24].

The channel is divided into nonoverlapping intervals through the division of channel-related parameter ranges, and each interval of selected parameters represents a state in FSMC model. The relevant parameter used in FSMC may be Signal-to-Noise Ratio (SNR) amplitude of received signal at the receiving end or collected energy. SNR can be selected as a parameter that composes SNR model [25]. The SNR of receiving end is divided into *K* levels, and each level is associated with a state of Markov chain. The block fading channel is considered to be that the SNR of receiving end is a constant within a period of time but will change according to Markov transition probability between different periods. Assume that random variable *γ* is the SNR of receiving end of terminal device *n*. That is, *γ* can be improved according to Markov chain of finite states, and all its states can be expressed as *κ*={1,2,…, *K*}. The realization of random variable *γ* of terminal device *n* in the time period *t* is represented as Γ(*t*), specifically expressed as

(1) $$\begin{array}{c} \Gamma_{n}\left(t\right)=k,\text{if }\gamma_{n}\left(t\right)\in \left.\left[h^{i-1},h^{k}\right.\right)\text{,} \end{array}$$

where *k* ∈ *κ*={1,2,…, *K*} and *h*^0^=0 < *h*^1^ < *h*^2^ < …. Let *ρ*_*s*_*n*_′*s*_*n*_^″^_(*t*) denote the probability of state Γ_*n*_(*t*) transitioning from state *s*_*n*_′ to state *s*_*n*_^″^ in the time period *t*. The *K* × *K* channel state transition probability matrix of terminal device *n* is denoted as Φ_*n*_(*t*)=[*ρ*_*s*_*n*_′*s*_*n*_^″^_(*t*)]_*K*×*K*_, where *ρ*_*s*_*n*_′*s*_*n*_^″^_(*t*)=Pr (Γ_*n*_(*t*+1)=*s*_*n*_′|Γ_*n*_(*t*)=*s*_*n*_^″^), *s*_*n*_′, and *s*_*n*_^″^ ∈ *κ*.

In practical applications, the transfer matrix can be observed and measured from wireless environment in the past. In addition, it is considered that {Γ_*n*_(*t*), 1 ≤ *t* ≤ *T*} exists independently for terminal device *n*. Based on FSMC channel model, Γ_*n*,*m*_ is used here to represent SNR between terminal device *n* and MEC server *m*. Since there is no interference between terminal devices, its channel efficiency can be expressed as *ϑ*_*n*,*m*_=log_2_ (1+Γ_*n*,*m*_). Considering that the bandwidth *W*_*m*_ of MEC server *m* is divided into *W*_*m*_/*B*_*m*_, the bandwidth of each channel is *B*_*m*_. Assuming that each user is allocated at most one channel, the transmission rate from terminal device *n* to MEC server *m* can be expressed as

(2) $$\begin{array}{c} v_{n,m}\left(t\right)=B_{m}\vartheta_{n,m}\left(t\right)\text{.} \end{array}$$

The subchannel owned by MEC server *m* has certain restrictions on receiving *W*_*m*_/*B*_*m*_; that is, the bandwidth allocated by MEC server *m* to all connected users cannot exceed the total bandwidth of MEC server *m*. Besides, MEC server is limited by cache and computing capacity. On the one hand, MEC server can only handle a limited number of tasks; on the other hand, the load that MEC server can handle is also limited (such as the number of computing tasks). Therefore, some tasks will be further offloaded to the core network to be processed by the core network. Use *g*_*u*_(*t*) ∈ {0,1} to represent the computation offloading decision indicator, which is used to indicate the way the server provides services. Among them, *g*_*n*_(*t*)=0 means that the terminal device *n* is processed by connected MEC server for computing tasks. And *g*_*n*_(*t*)=1 indicates that the task will be further offloaded to core network for processing by connected MEC server.

In order to further offload tasks to cloud, the wired backhaul link from MEC server to core network is considered. Assuming that the backhaul link capacity of network is *Z* (in bits per second), the backhaul link capacity allocated by MEC server *m* is *Z*_*m*_. Then, the following restrictions must be met:

(3) $$\begin{array}{c} \overset{N}{\underset{n=1}{\sum }}g_{n}\left(t\right)\theta_{n,m}\left(t\right)\varpi_{n,m}\left(t\right)\leq Z_{m}\text{,}\\ \overset{M}{\underset{m=1}{\sum }}\overset{N}{\underset{n=1}{\sum }}g_{n}\left(t\right)\theta_{n,m}\left(t\right)\varpi_{n,m}\left(t\right)\leq Z\text{,} \end{array}$$

where *θ*_*n*,*m*_ is the connection between terminal device *n* and MEC server *m* and *ϖ*_*n*,*m*_ is the transmission rate between terminal device *n* and MEC server *m*.

The sum of the rates of offloading computation tasks to the terminal device of core network by MEC server *m* cannot exceed the backhaul capacity of MEC server *m*. And the sum of speeds of all terminal devices processing computing tasks in the cloud cannot exceed the total backhaul capacity of system.

### 3.2. Calculation Model

If *G*_*n*_ is processed locally, use *T*_*n*_^*L*^ to represent the time when *G*_*n*_ is executed locally, which is specifically defined as

(4) $$\begin{array}{c} T_{n}^{L}=\frac{\phi_{n}}{f_{n}^{L}}\text{,} \end{array}$$

where workload *ϕ*_*n*_ is the total number of CPU cycles required to complete *G*_*n*_ and *f*_*n*_^*L*^ is the local computing power of terminal device *n* (i.e., the number of CPU cycles executed per second).

Use *E*_*n*_^*L*^ to represent the energy consumption of devices executed locally by *G*_*n*_, which is defined as follows:

(5) $$\begin{array}{c} E_{n}^{L}=\phi_{n}\times e_{n}\text{,} \end{array}$$

where *e*_*n*_ is terminal device *n* to calculate the energy consumption per unit of CPU cycle, *e*_*n*_=(*f*_*n*_^*L*^)^2^ × 10^−27^.

If *G*_*n*_ is processed at the edge, delay *T*_*n*_^*O*^ and device energy consumption *E*_*n*_^*O*^ under *G*_*n*_ edge execution should be calculated from three parts: data upload, data processing, and data return [26]. The specific calculation is as follows.

First, terminal device *n* uploads data *G*_*n*_ to the corresponding MEC server by wireless channel. Let *T*_*n*_′ be the time when device *n* uploads *G*_*n*_ data, which is defined as

(6) $$\begin{array}{c} T_{n}^{\prime }=\frac{D_{n}}{v^{\prime }}\text{,} \end{array}$$

where *D*_*n*_ is the data size of *G*_*n*_ and *v*′ is data upload rate in the system model (i.e., the amount of data uploaded per second).

Then, the energy consumption *E*_*n*_′ of terminal device *n* uploading data is

(7) $$\begin{array}{c} E_{n}^{\prime }=T_{n}^{\prime }\times P^{\prime }\text{,} \end{array}$$

where *P*′ is the uplink transmission power of terminal device *n*.

Then, MEC allocates computing resources for calculation after receiving processed data. Use *T*_*n*_^″^ to represent the time when the offloading data is calculated in MEC server, which is defined as

(8) $$\begin{array}{c} T_{n}^{\prime \prime }=\frac{\phi_{n}}{f_{nm}^{O}}\text{,} \end{array}$$

where *f*_*nm*_^*O*^ are the computing resources allocated by *m* MEC servers for *G*_*n*_ offload execution (i.e., the number of CPU cycles executed per second). When *G*_*n*_ is unloaded to the local or other MEC server, *f*_*ij*_^*O*^ is zero and serves as a constraint in the model, namely,

(9) $$\begin{array}{c} f_{nm}^{O}=0,x_{n}\neq m\text{.} \end{array}$$

At this time, terminal device *n* has no computing task and is in a waiting state and generates idle energy consumption. Suppose *P*_*n*_^*I*^ is the idle power of terminal device *n*, then the idle energy consumption *E*_*n*_^″^ of terminal device *n* under offloading computation is

(10) $$\begin{array}{c} E_{n}^{\prime \prime }=T_{n}^{\prime \prime }\times P_{n}^{I}\text{.} \end{array}$$

Finally, MEC server returns the calculation result to terminal device *n*. The calculation result during backhaul is small and downlink rate is high, so the time delay and energy consumption when terminal device is received are ignored. Therefore, delay *T*_*n*_^*O*^ under *G*_*n*_ edge execution is the sum of transmission delay *T*_*n*_′ and the calculation delay *T*_*n*_^″^ of MEC server, namely,

(11) $$\begin{array}{c} T_{n}^{O}=T_{n}^{\prime \prime }+T_{n}^{\prime }\text{.} \end{array}$$

The device energy consumption *E*_*n*_^*O*^ under *G*_*n*_ edge execution is the sum of upload energy consumption *E*_*n*_^″^ of device *n* and the idle energy consumption *E*_*n*_^″^ of device *n* waiting for *G*_*n*_ to complete calculation on MEC server, namely,

(12) $$\begin{array}{c} E_{n}^{O}=E_{n}^{\prime }+E_{n}^{\prime \prime }\text{.} \end{array}$$

In summary, the time delay *T*_*n*_ and energy consumption *E*_*n*_ of the entire calculation process of task *G*_*n*_ in terminal device *n* are

(13) $$\begin{array}{c} T_{n}=\left\{\begin{array}{cc} T_{n}^{L}\text{,} & x_{n}=0\text{,}\\ T_{n}^{O}\text{,} & x_{n}\neq 0\text{,} \end{array}\right.\\ E_{n}=\left\{\begin{array}{cc} E_{n}^{L}\text{,} & x_{n}=0\text{,}\\ E_{n}^{O}\text{,} & x_{n}\neq 0\text{.} \end{array}\right. \end{array}$$

Note that *T*_*n*_ and *f*_*nm*_^*O*^ should meet the following restrictions:

(14) $$\begin{array}{c} T_{n}\leq \eta_{n}\text{,}\\ T_{n}\leq \eta_{n}\text{.} \end{array}$$

The time delay constraint *η*_*n*_ of *G*_*n*_ is that computing power is twice 1.4 GHz. *F*_*m*_ is the overall computing resources of MEC server *m*; that is, the sum of computing resources allocated by each *G*_*n*_ that is offloaded to MEC server *m* should not exceed *F*_*m*_.

### 3.3. Problem Model

The purpose of this paper is to jointly optimize offloading decision-making and resource allocation scheme in the multiuser multi-MEC server scenario, considering the limited computing resources and time delay constraint of computing tasks. This allows all computing tasks to shorten the completion time and minimize energy consumption of all terminal devices while meeting the delay constraints and extend the use time of terminal devices [27, 28]. Thus, the system objective function Ψ is defined as

(15) $$\begin{array}{c} \Psi =\overset{N}{\underset{n=1}{\sum }}E_{n}+10\times \overset{N}{\underset{n=1}{\sum }}\frac{T_{n}}{\eta_{n}}\text{.} \end{array}$$

(*T*_*n*_/*η*_*n*_) is the ratio of completion time *G*_*n*_ to the delay constraints. According to the calculation results of simulation experiment, the difference between ∑_*n*=1_^*N*^*E*_*n*_ and ∑_*n*=1_^*N*^(*T*_*n*_/*η*_*n*_) is a decimal order of magnitude. Therefore, to ensure that the two are of the same order of magnitude and optimized together, ∑_*n*=1_^*N*^(*T*_*n*_/*η*_*n*_) is multiplied by a factor of 10. The objective function Ψ minimizes the ratio of overall energy consumption of terminal devices to the task execution time and delay constraints by solving the optimal offloading decision and resource allocation plan to achieve research purpose. The overall problem model is as follows:

(16) $$\begin{array}{c} \min_{X,f}\left(\Psi \right)\text{,}\\ X=\left[x_{1},x_{2},\ldots ,x_{n},\ldots ,x_{N}\right]\text{,}\\ X=\left[x_{1},x_{2},\ldots ,x_{n},\ldots ,x_{N}\right]\text{,}\\ y_{n}=\left\{\begin{array}{c} f_{n}^{L},x_{n}=0\text{,}\\ f_{nm}^{O},x_{n}=m\text{,} \end{array}\right.\\ s.t\text{.}\\ C_{1}:x_{n}\in \left\{0,1,\ldots ,m,\ldots ,M\right\},\forall n\in U\text{,}\\ C_{2}:y_{n}>0,\forall n\in U\text{,}\\ C_{3}:f_{nm}^{O}=0,x_{n}\neq m\text{,}\\ C_{4}:T_{n}\leq \eta_{n},\forall n\in U\text{,}\\ C_{5}:\overset{N}{\underset{n=1}{\sum }}f_{nm}^{O}\leq F_{m},\forall m\in H\text{,}\\ C_{6}:\left\{\begin{array}{c} \overset{N}{\underset{n=1}{\sum }}g_{n}\left(t\right)\theta_{n,m}\left(t\right)\varpi_{n,m}\left(t\right)\leq Z_{m}\text{,}\\ \overset{M}{\underset{m=1}{\sum }}\overset{N}{\underset{n=1}{\sum }}g_{n}\left(t\right)\theta_{n,m}\left(t\right)\varpi_{n,m}\left(t\right)\leq Z, \end{array}\right. \end{array}$$

where *X* is the task offloading decision amount and *Y* is the calculation resource allocation amount. Constraints *C*_1_, *C*_2_, and *C*_3_ indicate that each task *G*_*n*_ can only be offloaded to the local or one of MEC servers for calculation. *C*_4_ represents the constraint of task completion delay, and *C*_5_ and *C*_6_ represent the constraint that allocated computing resources should meet.

## 4. New Computation Offloading Method Based on Improved DQN

### 4.1. Multiagent Reinforcement Learning Algorithm

**Figure 2:**
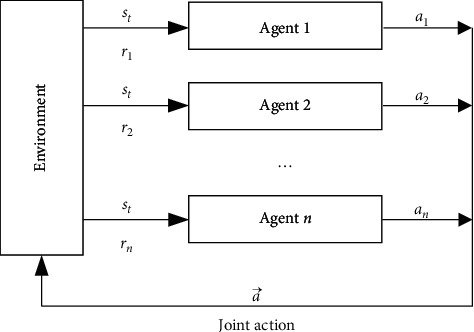
Multiagent reinforcement learning system.

The multiagent reinforcement learning system is shown in Figure 2, where multiple agents act at the same time. Under the joint action, the entire system will be transferred, and each agent will be rewarded immediately [29, 30].

For multiagent reinforcement learning, it is first necessary to establish a Markov game model. Markov game can be described by a multigroup (*n*, *S*, *A*_1_,…, *A*_*n*_, *R*_1_,…, *R*_*n*_). Among them,

(1) *n* is the number of agents; that is, *N* is the number of terminal devices. *S* is the system state, which generally refers to the joint state of multiple agents, that is, the joint state of each agent. The terminal device shares the current load status of edge computing servers, which can be expressed as
  (17) $$\begin{array}{c} LD\left(t\right)=\left[LD_{1}\left(t\right),LD_{2}\left(t\right),\ldots ,LD_{m}\left(t\right)\right]\text{,} \end{array}$$
where *LD*_*m*_ is the load of MEC.
(2) *R*_*i*_ is the instant reward function of each agent. That is, in current state *s*, after joint action (*A*_1_,…, *A*_*n*_) taken by multiple agents, the reward is obtained in the next system state $\hat{s}$.

The reward function completely describes the relationship between multiple agents. When the reward function of each agent is the same, that is, *R*_1_=*R*_2_=⋯=*R*_*n*_, it means that the agent is a complete cooperative relationship. When there are only two agents and reward function is opposite, that is, *R*_1_=−*R*_2_, it means that the agents are in perfect competition. When the return function is between the two, it is a mixed relationship between competition and cooperation.

### 4.2. Problem Description and Modeling

#### 4.2.1. Network Status

*S*={*s*(*t*)} represents the network state space, where *s*(*t*) represents the network state at time period *t*, and improvements are made in the entire time period *T*. The network status consists of SNR of each terminal device and cache status of each MEC server. *s*(*t*) can be defined as

(18) $$\begin{array}{c} s\left(t\right)=\left(\Gamma_{1}\left(t\right),\ldots ,\Gamma_{n}\left(t\right),\ldots ,\Gamma_{N}\left(t\right)\right.\text{,}\\ \left.\psi_{1}\left(t\right),\ldots ,\psi_{m}\left(t\right),\ldots ,\psi_{M}\left(t\right)\right)\text{,} \end{array}$$

where Γ_*n*_={Γ_*n*,*m*_, *m* ∈ *M*} represents SNR between user terminal device *n* and all MECs. *ψ*_*m*_(*t*)={*φ*_*k*,*m*_, *k* ∈ *K*} represents the cache status of MEC servers.

#### 4.2.2. Network Behavior

The intelligent agent needs to determine the attachment relationship between the terminal device and MEC server in each time period. That is the determination of the terminal device's computing offload, the allocation of computing resources, and the service cache policy of each MEC server. Thus, each executable action of terminal devices in the time period *t* can be defined as follows:

(19) $$\begin{array}{c} a\left(t\right)=\left(A_{1}\left(t\right),\ldots ,A_{n}\left(t\right),\ldots ,A_{N}\left(t\right)\right.\text{,}\\ G_{1}\left(t\right),\ldots ,G_{n}\left(t\right),\ldots ,G_{N}\left(t\right)\text{,}\\ \left.\psi_{1}\left(t\right),\ldots ,\psi_{m}\left(t\right),\ldots ,\psi_{M}\left(t\right)\right)\text{,} \end{array}$$

where *A*_*n*_(*t*) = {*a*_*n*,*m*_(*t*), *m* ∈ *M*} represents the attachment indicator of terminal device *n* and *G*_*n*_(*t*) represents the calculation and offloading decision of terminal device *n*.

#### 4.2.3. Reward Function

The goal is to maximize total benefit of system, but the reward function should be set to current benefit of system. First calculate the system leased spectrum and backhaul resources and allocate them to terminal devices part of the revenue. The unit price of spectrum leased from MEC server *m* is set to *δ*_*m*_ per Hz, and the unit price of backhaul link from MEC server *m* to core network is set to *σ*_*m*_ per bps. Corresponding to this, the calculation data is transmitted to MEC server corresponding to terminal device *n* and backhaul link from MEC server to the core network is used for charging. The unit price is defined as *α*_*n*_ per Hz and *β*_*n*_ per bps. Therefore, by summarizing this part of the income and expenditure, part of income for leased spectrum and backhaul resources obtained by terminal device *n* can be obtained:

(20) $$\begin{array}{c} R_{n}^{\prime }\left(t\right)=\alpha_{n}\overset{M}{\underset{m=1}{\sum }}a_{n,m}\left(t\right)B_{m}+\beta_{n}g_{n}\left(t\right)\overset{M}{\underset{m=1}{\sum }}a_{n,m}\left(t\right)R_{n,m}\left(t\right)-\overset{M}{\underset{m=1}{\sum }}\delta_{m}a_{n,m}\left(t\right)B_{m}-g_{n}\left(t\right)\overset{M}{\underset{m=1}{\sum }}\sigma_{m}a_{n,m}\left(t\right)R_{n,m}\left(t\right)\text{.} \end{array}$$

Then, calculate the profit obtained by terminal devices from allocating computing resources. On the one hand, when MEC side performs computing tasks, it needs to pay communication company for the loss of processing computing tasks and define the unit price of MEC server *m* energy consumption as *χ*_*m*_. On the other hand, the terminal device needs to pay a certain price for the server on MEC side, and computing resource allocated for each unit computing task is set to *ζ*_*n*_. Therefore, the benefit obtained by allocating computing resources to terminal device *n* can be calculated as

(21) $$\begin{array}{c} R_{n}^{\prime \prime }\left(t\right)=\left(1-d_{n}\left(t\right)\right)\overset{M}{\underset{m=1}{\sum }}\alpha_{n,m}\frac{\zeta_{n}F_{n,m}\left(t\right)}{L_{u_{n}}-\chi_{m}E_{n,m}^{MEC,e}\left(t\right)}\text{.} \end{array}$$

The amount of computing resources allocated to each unit computing task by the above formula has a very important impact on the completion time of computing task. Thus, the service cache cost mainly includes two parts: the cost of replacing type of cache supported on MEC side, and the cost of caching specific services on MEC server. Define the unit price of replacing cache type on MEC server *m* as *ξ*_*m*_ for each service type, and the unit price for caching services on MEC server is *ξ*_*m*_ per storage space. In order to increase the benefits of cache, the business type is quantified by weak backhaul from MEC server to the core network, which will be used to measure the cost of users. The benefits obtained by executing the cache service on MEC server *m* can be expressed as

(22) $$\begin{array}{c} R_{m}^{\prime \prime }\left(t\right)=\overset{N}{\underset{n=1}{\sum }}\beta_{n}\left(1-g_{n}\left(t\right)\right)R_{n,m}\left(t\right)-\xi_{m}\left|I\left[\psi_{m}\left(t\right)-\psi_{m}\left(t-1\right)\right]\right|-\varsigma_{m}\kappa \left|\psi_{m}\left(t\right)\right|\text{,} \end{array}$$

where |*ψ*_*m*_(*t*)| represents the number of nonzero elements, *I*[·] is an auxiliary function, and when *x* > 0, *I*(*x*)=1; otherwise, *I*(*x*)=0. The instant reward is designed as the total income of MVNO of all current users of system during the time period *t*, namely,

(23) $$\begin{array}{c} r\left(t\right)=\overset{N}{\underset{n=1}{\sum }}\left(R_{n}\left(t\right)+R_{n}^{\prime \prime }\left(t\right)\right)+\overset{M}{\underset{m=1}{\sum }}R_{m}^{\prime \prime }\left(t\right)\text{.} \end{array}$$

Here the long-term return *ℜ*(*t*) is expressed as

(24) $$\begin{array}{c} R\left(t\right)=\overset{T}{\underset{t=1}{\sum }}\epsilon r\left(t\right)\text{,} \end{array}$$

where *ϵ* ∈ [0,1) is the discount rate of future earnings weights. When *ϵ* approaches 1, the system will pay more attention to long-term benefits, and when *ϵ* approaches 0, the system will pay more attention to short-term benefits.

### 4.3. Dueling-DQN

DQN is an effective reinforcement learning algorithm, which can make the agent learn good experience from the interaction with environments [31–33]. At the same time, according to DQN learning mechanism, there are improvements to DQN algorithm in different aspects. In DQN, due to the error in the Q estimated value itself, max_*a*_ *Q* process can be seen according to the expression. It is equivalent to putting forward the largest error, which also leads to the problem of overestimation. Double-DQN is an effective improved algorithm for this problem. In Double-DQN algorithm, the update form of $\hat{Q}\left(S\right)$ is changed to

(25) $$\begin{array}{c} \hat{Q}\left(s\right)=R\left(s\right)+\lambda \cdot \hat{Q}\left(\hat{s},\max_{a}Q_{\text{eval}}\left(\hat{s},a;\alpha \right);\alpha^{-}\right)\text{,} \end{array}$$

where *λ* is the discount factor.

The Double-DQN algorithm takes advantage of double neural network and uses two neural networks to learn at the same time, effectively avoiding the overestimation problem caused by error amplification.

**Figure 3:**
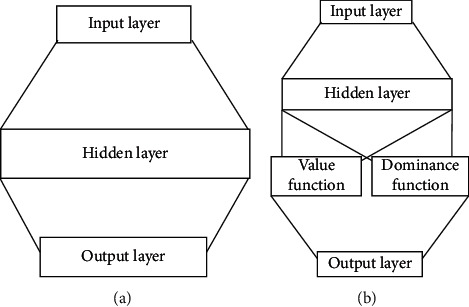
Comparison between DQN algorithm and Dueling-DQN algorithm. (a) DQN. (b) Dueling-DQN.

Dueling-DQN is also an improvement to DQN algorithm. Compared with previous algorithms, Dueling-DQN algorithm learns faster and has better results. Compared with DQN algorithm, Dueling-DQN retains most of the learning mechanism, and the only difference is the improvement of neural network, as shown in Figure 3.

In the traditional DQN algorithm, the output result is Q value corresponding to each action. In Dueling-DQN algorithm, the output is expressed as a combination of two parts: the value function and advantage function [34]. Among them, value function refers to the value of a certain state, and advantage function refers to the advantage obtained by each action on the state. Therefore, in Dueling-DQN algorithm, Q value problem in DQN can be reexpressed as the following form:

(26) $$\begin{array}{c} Q\left(s,a;\alpha ,\omega_{1},\omega_{2}\right)=V\left(s;\omega ,\omega_{2}\right)+l\left(s,a;\omega ,\omega_{1}\right)-\frac{1}{\left|l\right|}\sum_{a^{\prime }}l\left(s,a^{\prime };\omega ,\omega_{1}\right)\text{,} \end{array}$$

where *V*(·) and *ℓ*(·) are the value function and advantage function, respectively, and *ω* is the parameter of neural network convolutional layer. *ω*_1_, *ω*_2_ are the parameters of two control flow layers, respectively. The latter item of the plus sign centralizes the advantage function in order to solve the uniqueness problem of Q value.

## 5. Experimental Results and Analysis

The specific simulation parameters are as follows.

Assume that the computing power of each device *n* is 1.5 GHz, the uplink transmission power is 800 mW, the idle power is 100 mW, and the upload rate is 2.5 Mb/s. *M* = 4 and overall computing capacity of each MEC server is 6 GHz, 5 GHz, 3 GHz, and 1 GHz, respectively. The data *D*_*n*_ in task *G*_*n*_ obeys uniform distribution of (600, 1200), and the unit is k bits. The workload *ϕ*_*n*_ obeys uniform distribution of (1000, 1500), and the unit is Megacycles.

For the parameters of Dueling-DQN algorithm, set the learning rate *ϵ* to 0.001 and discount coefficient *λ* to 0.90. The size of experience replay set is 3000, and the number of randomly sampled samples is 40.

### 5.1. Parameter Analysis

#### 5.1.1. Learning Rate Analysis

**Figure 4:**
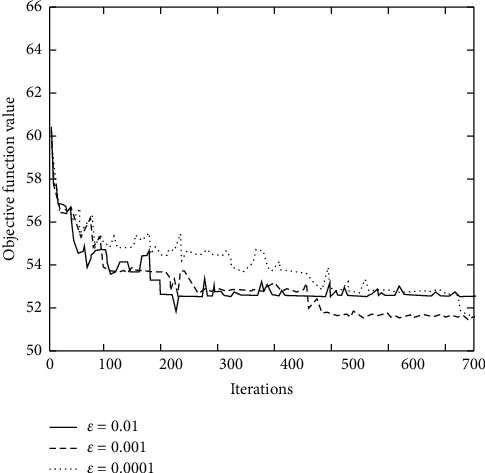
Convergence of the proposed algorithm under different learning rates.

The learning rate of the algorithm will have a great impact on the performance of the proposed strategy. Therefore, three different learning rates *ϵ* of 0.01, 0.001, and 0.0001 are selected to compare the convergence of improved DQN algorithm, as shown in Figure 4.

#### 5.1.2. Discount Factors Analysis

**Figure 5:**
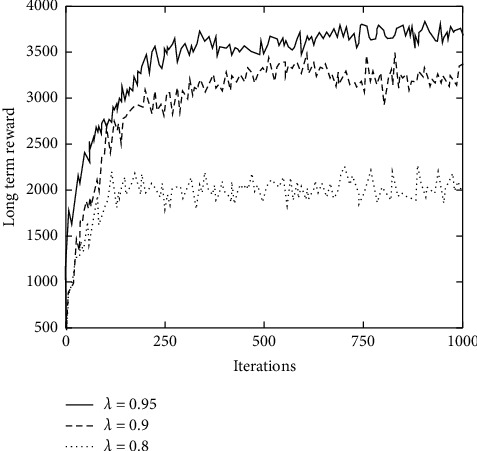
System long-term rewards with different discount rates.

Similarly, the influence of discount factor on improved DQN algorithm is shown in Figure 5, where the discount factor takes values 0.8, 0.9, and 0.95.

It can be seen from Figure 5 that as the discount factor increases, the long-term reward is continuously increasing. When *λ* is 0.95, the long-term reward is 3700 when it is stable. Because the discount factor will affect behavior selection strategy, that is to say, a larger discount factor will cause system to pay more attention to long-term benefits, and a lower discount factor will cause system to pay more attention to current benefits, a higher discount factor will often lead to greater long-term benefits. However, in actual use, using an overly high discount factor does not have corresponding benefits. This is because the system in reality is more changeable, and too much emphasis on future benefits will lead to excessive calculations and excessive losses in the system, which often requires a trade-off.

### 5.2. Optimization Comparison under Different Objective Functions

For multiobjective optimization problems that reduce time delay and energy consumption, the weighted sum of task execution time delay and terminal execution energy consumption is usually used as the objective function to solve problem, and the calculation is as follows:

(27) $$\begin{array}{c} \Psi^{\prime }=\frac{\sum_{n=1}^{N}\left(\omega_{t}\times T_{n}+\left(1-\omega_{t}\right)\times E_{n}\right)}{N}\text{,} \end{array}$$

where *ω*_*t*_ is the weight coefficient of execution delay and 1 − *ω*_*t*_ is the weight coefficient of execution energy.

Comparing (22) with the proposed objective function (13) to optimize the delay and energy consumption, the number of terminal devices is 12. Considering that the goal of the proposed strategy is to shorten time delay and reduce energy consumption while satisfying the time delay constraints, therefore, the values of *ω*_*t*_ are, respectively, 0.8, 0.6, and 0.4, and the joint experiments of Energy Reduced Scale (ERS) and Time Reduced Scale (TRS) are carried out, as shown in Table 1.

It can be seen from Table 1 that when *ω*_*t*_ is 0.8 and 0.6, the control strategy pays more attention to the optimization of time delay, and when *ω*_*t*_ is 0.4, optimization results pay more attention to the optimization of energy consumption. However, the optimization result of the proposed objective function is the best, and ERS and TRS are 52.18% and 34.72%, respectively, which can shorten time delay and reduce energy consumption under the time delay constraints.

When computing task is 150, comparing control strategies under the four objective functions with the random offloading strategy, the results of ratio of the time delay and energy consumption reduction are shown in Table 2.

It can be seen from Table 2 that delay and energy consumption optimization effect of the proposed optimization target is better, and the reduction ratio of delay and energy consumption is 2.58% and 30.67%, respectively, because the optimization objective of the proposed strategy comprehensively considers the offloading decision and resource allocation plan of joint optimization system when the computing resources are limited and computing tasks have time delay constraints. This allows all computing tasks to shorten completion time and minimize the energy consumption of all terminal devices while meeting the delay constraints. This demonstrates the effectiveness of the proposed objective function.

### 5.3. Performance Comparison with Other Algorithms

In order to demonstrate the performance of the proposed strategy, compare it with [12], [19], and [14] in terms of objective function value, calculation amount, and time saving. Li and Jiang [12] proposed a distributed task offloading strategy, which selects the best MEC node offloading amount by game equation on the basis of quantifying offloading cost and delay. Reference [14] used the greedy selection algorithm to design the maximum energy-saving priority algorithm and energy priority strategy to achieve optimal offloading of computing tasks on mobile devices. Reference [19] used the time average calculation rate maximization algorithm to jointly and efficiently allocate radio resources and computing resources.

#### 5.3.1. Algorithm Comparison under Different Cumulative Tasks

**Figure 6:**
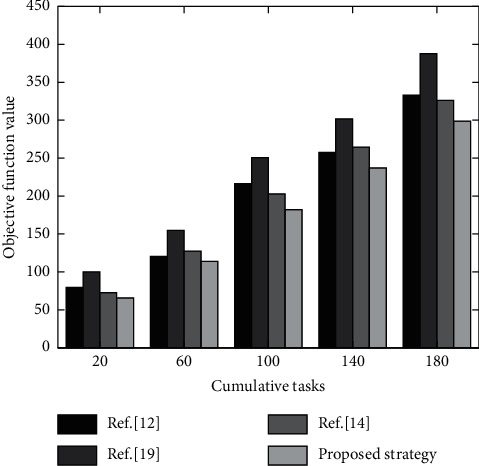
Comparison results of offloading strategies under different cumulative number of tasks.

In the experiment, objective function value results of the four strategies are shown in Figure 6 for different accumulations of computing tasks.

It can be seen from Figure 6 that the value of objective function is gradually increasing with the increase of cumulative number of tasks for the four offloading strategies. However, the proposed strategy has a relatively lower objective function value than other strategies. That is, the energy consumption and delay are relatively small. For example, when the number of tasks is 180, the objective function value is only 298. Since the proposed strategy considers computation offloading and resource allocation comprehensively, improved deep learning algorithm is used for optimization, and delay and energy consumption are minimized. Reference [19] only matched computing resources but did not rationally optimize the task offloading scheme and computing resource allocation scheme, resulting in high task execution time delay and energy consumption. References [12] and [14] both used corresponding algorithms for optimization to achieve better resource allocation and task offloading. But their analysis of time delay is less, so the performance needs to be strengthened.

#### 5.3.2. Computation Number Comparison of Offloading Tasks under Different Offloading Strategies

**Figure 7:**
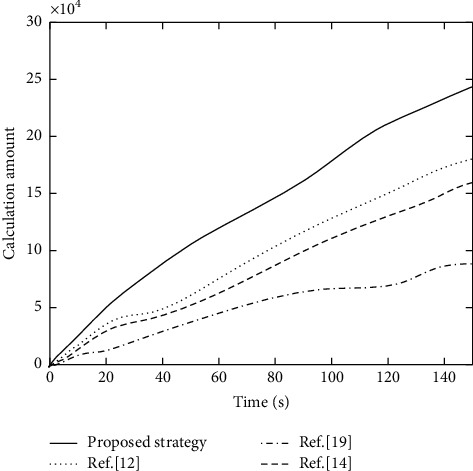
Comparison results of the computation number of offloading tasks under different offloading strategies.

Under four different computation offloading strategies, the comparison results of the computing number of offloading tasks on terminal device side are shown in Figure 7. Vertical axis represents the total calculation number of tasks performed by all terminal devices to perform calculation and offloading. The calculation number of tasks is used to represent the amount of calculation services provided by MEC server. Therefore, the evaluation indicators in the figure also represent the benefits of computing terminal devices in the offloading mode.

It can be seen from Figure 7 that as time increases, computing tasks continue to increase, and the amount of task calculations also increases. However, the calculation amount of the proposed strategy is significantly better than other comparison strategies. Taking the simulation time of 140 s as an example, compared with [12], [19], and [14], the proposed strategy has increased by 11.54%, 20.83%, and 152.72%, respectively. It can be argued that the proposed strategy is the best compared to task offloading. It uses Dueling-DQN algorithm to process task offloading and resource allocation models, and its optimization performance is better than the greedy selection algorithm in [14] and the game equation model in [12].

#### 5.3.3. Energy-Saving Comparison of per Unit Terminal Devices

**Figure 8:**
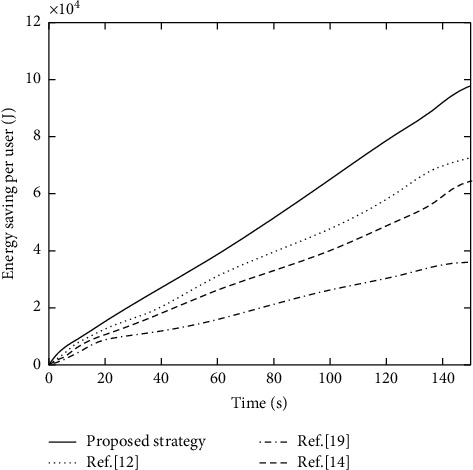
Comparison results of energy saving per unit terminal device under different offloading strategies.

Under four different computation offloading strategies, the comparison of energy consumption saved by each terminal device by computation offloading on average is shown in Figure 8. In the local calculation model, all energy consumption is generated by local calculations. In the computation offloading mode, the energy consumption is communication energy consumption caused by upload tasks. For the task of performing computation offloading, the difference between the two is energy saving.

It can be seen from Figure 8 that, compared with other comparison strategies, the proposed strategy has the largest energy-saving rate, which is close to 10 × 104 J; this also means the least energy consumption. Aiming at the overestimation problem in DQN, the proposed strategy uses Dueling-DQN algorithm for optimization. And it designs the system benefits and resource consumption as rewards and losses, which improves the efficiency and rationality of task offloading and resource allocation by optimizing problem solution. Reference [19] only used the time average calculation rate maximization algorithm to efficiently allocate computing resources. The optimization algorithm is more traditional and has poor performance. Thus, the overall energy saving is not high. Reference [12] used the game equation model to optimize task offloading strategy but does not realize the rationalization of resource allocation. Therefore, the maximum energy saving is 710 × 104 J. Reference [14] used greedy selection algorithm to design an optimal energy-saving strategy but did not consider server computing resource constraints and task delay constraints. Therefore, the overall performance is not as good as the proposed strategy.

## 6. Conclusion

MEC server has limited computing resources and computing task has delay constraint. How to shorten completion time and reduce terminal energy consumption under the delay constraints becomes an important research issue. To solve this problem, this paper proposes a task offloading and resource allocation strategy based on deep learning for MEC. In the multiuser multiserver MEC environment, a new objective function is designed to construct mathematical model. In combination with deep reinforcement learning, the partially improved Dueling-DQN algorithm is used to solve the optimization problem model, which can reduce the completion time of computing tasks and minimize energy consumption of all terminal devices under the delay constraints. The proposed strategy is demonstrated by experiments based on Python platform. The experimental results show that when learning rate is 0.001 and discount factor is 0.90, the energy saving is close to 10 × 104 J, which is better than other comparison strategies. In terms of calculation amount, it increased by 11.54%, 20.83%, and 152.72%, respectively.

In practice, different users have different concerns about service quality. Therefore, we can refer to the different needs of users when making computation and offloading decisions in the following research. It can assign a certain weight to the factors affecting the quality of service and combine the task priority for scheduling.

## Figures and Tables

**Table 1:** Comparison results of optimization for different objective functions.

Objective function	ERS (%)	TRS (%)
*ω*_*t*_ = 0.4	48.71	27.96
*ω*_*t*_ = 0.6	41.85	31.38
*ω*_*t*_ = 0.8	31.03	32.56
Formula (13)	52.18	34.72

**Table 2:** Comparison results of optimization for four objective functions.

Objective function	Delay reduction ratio (%)	Energy consumption reduction ratio (%)
*ω*_*t*_ = 0.4	1.25	23.29
*ω*_*t*_ = 0.6	1.97	21.16
*ω*_*t*_ = 0.8	2.03	19.98
Formula (13)	2.58	30.67

## Data Availability

The data used to support the findings of this study are included within the article.
